# The Institutional Worker

**Published:** 1912-07-13

**Authors:** 


### THE INSTITUTIONAL WORKER

**Being a Special Supplement to "The Hospital"**

### Editor's Notices.

**Contributions:**

Contributions should be written, or preferably typed, on one side of the paper only, and all articles sent in are accepted upon the distinct understanding that they are forwarded to The Hospital only.

The Editor cannot undertake to return MSS. not used, but when a stamped directed envelope is enclosed the MSS. may be returned if a special request is made.

Accepted articles and paragraphs of news will be paid for after publication at the scale rate.

**Address:**

To prevent delay all contributions and letters on editorial business must be addressed exclusively to the Editor, "The Hospital," 29 Southampton Street, Strand, London, W.C. It is important that this regulation shall be strictly observed.

**Correspondence:**

Correspondence on all subjects is invited. The name and address of correspondents must be given as a guarantee of good faith, but not necessarily for publication.

**Special Articles:**

Special articles are invited, and questions, inquiries, and paragraphs upon all matters relating to the work, administration, and management of general, special, mental, fever, cottage and convalescent hospitals, sanatoria, homes, institutions, societies and organisations for the treatment or care of the sick, injured and dependants of all classes. Every matter affecting the interests and welfare of the staff of all grades working in these institutions will receive special consideration.

**Administrative Medicine, Research and Items of News:**

Special terms will be paid for approved articles on subjects in this wide field by experts who have made some section of it their special study and interest. We wish to give prominence to every movement tending to advance accuracy of diagnosis, efficiency in treatment, and the development of modern methods to eradicate disease and promote the welfare of the suffering.

**A Bureau of Information:**

We invite inquiries and applications for information and help in providing for that numerous class of sufferers who require special care or house-room which they cannot obtain in their own homes or secure for themselves. **We cannot, however, prescribe, or recommend practitioners.**

**Books for Review:**

Publishers are particularly requested to send advance proofs of any new books of importance whenever possible, as the Editor has made arrangements to publish immediate reviews on a new plan.

**Photographs, Plans, Blocks, and Illustrations:**

It is requested that wherever possible MSS. may be accompanied by illustrations in any of the above forms. The name of the sender and of the article to which it belongs should be written on the back of each photograph, drawing, or block for purposes of identification.

**Local Papers:**

Newspapers containing reports or news paragraphs **should be marked** and addressed to the Sub-Editor.

**The Coupon System:**

A coupon will be found at the bottom of the third inside page of the cover each week. This coupon must be attached to every question or inquiry to which an answer is desired in The Hospital.

### The Greater Problem.

Where to turn for information on the many problems that frequently present themselves to the Institutional Worker ofttimes itself becomes the greater problem, and the want of immediate help may result in indecision and confusion, which might have been avoided had the required knowledge been easily accessible. Consequently the conductors of The Hospital have instituted a

**BUREAU OF INFORMATION**

which provides the means whereby workers in every department of Institutional Life can obtain expert advice and practical assistance in the many difficulties that arise from time to time in connection with their work. The Bureau is open to every reader of the Journal, and enquiries on all subjects are cordially invited. As the queries are dealt with by those best qualified to answer the questions or to offer a solution to the difficulty, it will be appreciated that the new department must prove of the greatest service to everyone interested or associated with Institutional Work. To those of our readers who desire a fuller knowledge of the work of the Bureau we would advise them to send at once for the booklet which has been recently published explaining the scope of this new department. All communications should be addressed to The Hospital Bureau of Information, The Hospital Building, 28 and 29 Southampton Street, Strand, London, W.C.

### Manager's Notices.

**All letters on business matters must be addressed to The Manager, "The Hospital," 28 and 29 Southampton Street, London, W.C.**

**Advertisements:**

To ensure insertion, all advertisements for The Hospital must reach the Manager not later than Wednesday morning in each week. For scale of charges see pages v and 2.

**Subscriptions,** which may commence at any time, are payable in advance. Cheques and Post-Office Orders should be crossed London County and Westminster Bank, Covent Garden Branch, and made payable to the Manager of The Hospital.

Rates of Subscription (including Postage).

| *United Kingdom.* | | *Foreign and Colonial.* | |
|---|---|---|---|
| Three Months ... ... | 2s. 0d. | Three Months ... ... | 2s. 6d. |
| Six Months ... ... | 4s. 0d. | Six Months ... ... | 5s. 0d. |
| Twelve Months ... ... | 6s. 6d. | Twelve Months ... ... | 8s. 8d. |

*SUBSCRIPTION FORM.*

*Please send me* The Hospital *for.................months, commencing with your issue of.............................., for which please find enclosed............................*

*Name*..................................................

*Address* ..................................................

..................................................

*Date*......................................

*To* ...................................... *Newsagent.*

**Remittances:**

**It is especially requested that remittances be made by Cheque or Postal Order, and in halfpenny stamps only when the amount is under sixpence.**

### OFFICIAL APPOINTMENTS VACANT.

### Poor-Law Vacancies.

Assistant Cook (£26), Wandsworth Union. Mr. F. W. Piper, C. to G., St. John's Hill, Wandsworth, S.W. July 16.

Assistant Male Attendant (£25), Sunderland Union. Mr. W. P. Brantingham, C. to G., Sunderland. July 13.

Assistant Male Cook (£35), Brentford Union. Mr. W. Stephens, C. to G., Isleworth, W. July 17.

Assistant Nurse (£16), Stepney Union. Mr. T. G. Stacey, C. to G., Barnes Street, Stepney, E. July 17.

Barber and Bath Attendant (£33 18s.), St. George-in-the-East Guardians. Mr. R. M. Lochner, C. to G., Raine Street, Old Gravel Lane, E. July 16.

Charge Nurse (£35), Oldham Union. Mr. H. A. Quarmby, C. to G., Oldham. July 16.

Charge Nurse (£32), Newhaven Union. Mr. H. W. Coupe, C. to G., Newhaven. July 17.

Charge Nurses (£30), Loughborough Union. Mr. A. W. Jarratt, C. to G., Loughborough. July 13.

Children's Caretaker (£22), Warwick Union. Mr. C. H. Passman, C. to G., Leamington. July 15.

Cook (£35), Tonbridge Union. Mr. N. R. Stone, C. to G., Tunbridge Wells. July 16.

Cook, etc. (£30), Ripon Union. Mr. G. F. P. Edmundson, C. to G., Ripon. July 16.

Female Cook (£30), East Preston Union. Mr. A. Shelley, C. to G., Littlehampton. July 15.

Female Ambulance Attendant (£25), Leeds Union. Mr. J. H. Ford, C. to G., Leeds. July 13.

Female Attendant (£18), Chapel-en-le-Frith Union. Mr. J. B. Boycott, C. to G., Chapel-en-le-Frith. July 13.

Female Infirm Attendants (£25), Hampstead Guardians. Mr. H. W. Preston, C. to G., New End, Hampstead. July 16.

Foster-Mother (£25), Taunton Union. Mr. W. F. B. Dawe, C. to G., Taunton. July 20.

Head Nurse (£30), Croydon Union. Mr. H. List, C. to G., Thornton Heath, Surrey. July 16.

Head Nurse (£35), Redruth Union. Mr. T. Peter, C. to G., Redruth. July 16.

Infants' Nurse (£30), Bridgend and Cowbridge Union. Mr. R. H. Cox, C. to G., Bridgend. July 13.

Instructor in Domestic Economy (£85), South Manchester Guardians. Mr. D. S. Bloomfield, C. to G., All Saints, Manchester. July 23.

Labour Mistress (£25), Hampstead Guardians. Mr. H. W. Preston, C. to G., New End, Hampstead. July 16.

Laundress (£23), Long Ashton Union. Mr. A. E. Hicks, C. to G., Flax Bourton. July 15.

Laundry Superintendent (£20), Conway Union. Mr. J. W. Post, C. to G., Conway. July 19.

Male Attendant (£30), Cardiff Union. Mr. A. J. Harris, C. to G., Cardiff. July 19.

Master and Matron (£70 and £40), Bridgwater Union. Mr. T. M. Reed, C. to G., Bridgwater. July 16.

Nurse (£26), Todmorden Union. Mr. F. Hollinrake, C. to G., Todmorden. July 15.

Nurse (£25), Runcorn Union. Mr. G. F. Ashton, C. to G., Runcorn. July 15.

Nurse (£30), Merthyr Tydfil Union. Mr. F. T. James, C. to G., Merthyr Tydfil. July 18.

Nurse (£35), Sheppey Union. Mr. J. Copland, C. to G., Sheerness. July 23.

Nursery Attendants (£30), Prestwich Union. Mr. E. W. Ogden, C. to G., Cheetham Hill Road, Manchester. July 15.

Porter (£25), Bedford Union. Mr. W. Payne, C. to G., Bedford. July 22.

Seamstress and Relief Foster-Mother (£22), Hull Guardians. Mr. R. H. Winter, C. to G., Hull. July 16.

Temporary Nurse (£1 weekly), Williton Union. Mr. F. Risdon, C. to G., Williton, Somerset. July 13.

Third Assistant Clerk (£80), Paddington Guardians. Mr. H. F. Aveling, C. to G., 313-319 Harrow Road, W. July 22.

### Municipal Vacancies.

Assistant Inspector of Nuisances (£90), Chester T.C. Mr. J. H. Dickson, Town Clerk, Chester. July 13.

Assistant Inspector of Nuisances (30s. weekly), Cardiff T.C. Mr. J. L. Wheatley, Town Clerk, Cardiff. July 15.

Assistant Sanitary Inspector (£100), Southend-on-Sea T.C. Mr. C. G. Pugh, Medical Officer of Health, Southend-on-Sea. July 13.

Assistant Sanitary Inspector (£120), Southgate U.D.C. Mr. W. M. Ellenor, Clerk to the Council, Palmer's Green, N, July 13.

Assistant Sanitary Inspector (£125), Hornsey T.C. Mr. F. D. Askey, Town Clerk, Highgate, N. July 15.

Assistant Sanitary Inspector (£90), Nuneaton T.C. Mr. F. S. Clay, Town Clerk, Nuneaton. July 17.

Clerk and Chief Assistant Overseer (£250), Chislehurst U.D.C. Mr. P. W. Straus, Hatton Cottage, Chislehurst. July 20.

Clerk Assistant (£65), Doncaster T.C. Mr. D. L. Anderson, Medical Officer of Health, Doncaster. July 18.

District Highway Surveyor (£100), Newbury R.D.C. Mr. S. V. Pinniger, Clerk to the Council, Newbury. July 26.

Health Visitor (£80), Ilkeston T.C. Dr. M. H. Archibald, Town Hall, Ilkeston. July 15.

Highways Surveyor (state salary), Whittington and Newbold U.D.C. Mr. H. J. Watson, Clerk to the Council, 32 Gluman Gate, Chesterfield. July 13.

Hospital Caretakers (married couples), Alton Joint Hospital Committee. Mr. W. B. Trimmer, Clerk to the Committee, Town Hall, Alton. July 15.

Inspector of Nuisances and Surveyor (£200 thereabouts), Newport Pagnell R.D.C. Mr. C. W. Powell, Clerk to the Council, Newport Pagnell. July 13.

Nurse (£30), Wetherby R.D.C. Isolation Hospital. Mr. E. H. Coates, Clerk to the Council, Wetherby. July 13.

School Dentist (£220), Exeter T.C. Mr. H. L. Parry, Town Clerk, Exeter. July 15.

Surveyor (£150), Ripon T.C. Mr. M. Kirkley, Town Clerk, Ripon. July 15.

Town Clerk (£450), Luton T.C. Mr. Bruce Penny, Town Clerk, Luton. July 22.

